# Electrophoresis of polyelectrolyte‐adsorbed soft particle with hydrophobic inner core

**DOI:** 10.1002/elps.202400143

**Published:** 2024-09-17

**Authors:** Asim Mahata, Sanjib Kumar Pal, Hiroyuki Ohshima, Partha P. Gopmandal

**Affiliations:** ^1^ Department of Mathematics Jadavpur University Kolkata West Bengal India; ^2^ Faculty of Pharmaceutical Sciences Tokyo University of Science Noda Chiba Japan; ^3^ Department of Mathematics National Institute of Technology Durgapur Durgapur West Bengal India

**Keywords:** electrophoretic mobility, exponential distribution, flat‐plate regime, hydrophobicity, ion steric effect

## Abstract

This article deals with the electrophoresis of hydrophobic colloids absorbed by a layer of polymers with an exponential distribution of the polymer segments. The functional groups present in the polymer layer further follow the exponential distribution. We made an extensive mathematical study of the electrophoresis of such core‐shell structured soft particles considering the combined impact of heterogeneity in polymer segment distribution, ion steric effect, and hydrodynamic slippage of the inner core. The mathematical model is based on the flat‐plate formalism and deduced numerical results for electrophoretic mobility are valid for weak to highly charged particles for which the particle size well exceeds the Debye‐layer thickness. In addition, we have derived closed form analytical results for electrophoretic mobility of the particle under several electrohydrodynamic limits. We have further illustrated the results for electrophoretic mobility considering a charged and hydrophobic inner core coated with an uncharged polymer layer or a polymer layer that entraps either positive or negatively charged functional groups. The impact of pertinent parameters on the overall electrophoretic motion is further illustrated.

## 1 INTRODUCTION

Electrophoresis is an analytical technique to separate various macromolecules [1, 2]. Besides, such a phenomenon finds its potential application in various fields, for example, in paper display application [3], to fabricate thin films [4], to sequence DNA [5], in food processing industry [6], and targeted drug delivery systems [7], to name a few. It is noteworthy to mention the electrophoresis of colloidal entities refers to their motion under the influence of an applied electric field. When the charge colloidal entities are immersed in an electrolytic microenvironment, an electric double layer (EDL) forms next to the charged surface. The excess counterions within the EDL experience a net electromotive force under an applied electric field and induce the particle motion [8]. The classical representation of colloidal entities refers the particle to be rigid (impermeable to fluid flow and ions from electrolytic microenvironment) and for such a case, the electrophoretic mobility (i.e., electrophoretic velocity per unit field strength) depends on the surface ζ‐potential, the key indicator of electrical charges carried by the charged entities [9]. Thus, the electrophoresis technique is routinely employed to extract the quantitative information of ζ‐potential of rigid colloidal entities. However, such a rigid representation of colloidal particles is no longer applicable for various biocolloids and environmental entities, including virus [10], bacteria [11], humic acid [12], pollutant [13], bio‐functionalize nanoparticulates [14], to name a few. Such nanoparticulates and environmental entities are in fact core‐shell structured and are collectively termed as soft particles, which are composed of an inner rigid core and grafted with a polyelectrolytic layer (PEL). The peripheral shell layer allows the flow penetration of ionized fluid. The inner rigid core may bear nonzero surface charge as well as the peripheral PEL further entraps ionizable functional groups, which in turn leads to the formation of net nonzero volume charges within the PEL. For such nanoparticulates and environmental entities, the EDL, which forms along the surface of the inner core, further modified due to the presence of PEL‐charge as well as flow penetration of ionized fluid across the PEL. Thus, in principle, the conventional concept of ζ‐potential loses its meaning for the core‐shell structured nanoparticulates and environmental entities [15].

Due to the widespread applicability of the soft representation of biocolloids and environmental entities, several researchers studied the electrophoresis of such particles. Based on Debye–Bueche theory of hydrodynamics of polymer solutions [16], a series of studies are available on the electrophoresis of soft particles within flat‐plat formalism [17, 18, 19, 20, 21, 22, 23]. Such a representation is strictly valid for the case when the particle size well exceeds the EDL thickness. Later, Ohshima [24, 25, 26, 27, 28] made extensive contributions in this direction and provided closed form analytical results for soft particles considering the curvature effect of the particle, and various combinations of charge properties of the inner core as well as PEL. In addition, several other researchers extended aforesaid theoretical studies to consider the double layer polarization and relaxation effects on the electrophoresis of soft particle [29, 30, 31, 32]. Readers are further referred to the review articles for the recent development of electrohydrodynamics of soft particles [33, 34]. In all these studies, the ions are considered to be point‐like charge, and thus, allow the accumulation of a sufficiently large number of counterions near a moderate to highly charged inner core as well as within the PEL. Such a representation of ions as point‐like charge thus neglects the ion steric effect induced due to interaction of finite sized ions. Gopmandal et al. [35] studied the electrophoresis of soft particle considering ion steric effect. Based on the Carnahan–Stirling model [36], they [35] have deduced electrophoretic mobility within flat‐plat formalism. It is noteworthy to mention, the Carnahan–Stirling model [36] appears to provide a correct interpretation of the ion steric effect for the entire range of electrolyte concentration and electrostatic charge properties of the colloidal entities [37, 38]. Another important parameter that affect the electrophoresis of soft particle is the hydrophobic behavior of the inner core. The nanoparticulates in which the hydrophobic anticancer drug material enclosed inside a porous layer can be treated as a core‐shell structured soft particle with hydrophobic inner core [39]. As expected, such a composite nanoparticle finds their widespread applications in targeted drug delivery system. Note that the tangential velocity along the surface of the hydrophobic inner core is nonzero. Thus, the Navier‐slip velocity boundary condition needs to be considered to account the fluid velocity along the tangential direction of the surface of hydrophobic inner core [40]. Bharti et al. [41, 42] studied extensively the electrophoresis of such soft particles with hydrophobic inner core and they observed that the hydrodynamic slippage of the inner core has substantial impact on the overall electrophoretic motion.

All the studies on the electrophoresis of soft particles indicated above deal with the typical situation where the monomers as well as the volume charges are distributed uniformly across the PEL. However, there are various practical relevances where the distribution of monomers, thereby volume charge across the absorbed polymeric layer, may not always be uniform [43]. Considering the nonuniform distribution of monomers and accompanying volume charge across the PEL, a few research articles are available that deal with the electrophoretic motion of such colloids. Duval and Ohshima [44] studied electrophoresis of diffuse soft particles, where the monomer and volume charge across the PEL is modeled via sigmoidal function. Gopmandal et al. [45] further extend the work of Duval and Ohshima [44] to consider the impact of hydrophobicity of the inner core. Chowdhury et al. [46] studied the electrophoresis of soft particles, where the PEL is modeled via a semisoft function, which is certainly applicable for soft layers in which the PEL is semidiffuse in nature. Varoqui [47] mentioned that the monomer distribution across absorbed polymers often follow the exponential distribution and they studied the electrophoresis of core‐shell particles with a charged inner core coated with an uncharged absorbed polymer. Later Ohshima [48] extended the work of Varoqui [47] considering charged absorbed polymers. However, in their works [47, 48], the inner core is considered to be hydrophilic and they have neglected the impact of ion steric effect.

In this article, we consider the electrophoresis of soft particles where the inner core is coated with adsorbed polymers. The charged inner core is considered to be hydrophobic. The monomers and accompanying volume charge across the peripheral layer are made of absorbed polymer and are modeled via exponential function [47, 48]. The impact ion steric effect is further considered in our study, and the same effect is modeled via the Carnahan–Stirling model [36]. We perform our analysis in flat‐plate formalism. Herein, we have provided the mathematical results for electrophoretic mobility, which are certainly useful to measure the electrostatic charge properties as well as to capture the electrokinetic motion of soft colloids, which resembles various biocolloids and environmental entities (e.g., bacteria, viruses), as well as engineered nanoparticles. The rest of the article is organized as follows. In Section 2, we present the mathematical formulation of electrophoresis of the undertaken soft particle. The general expression for electrophoretic mobility and various closed form limits deduced under several limits are presented in Section 3. The discussion on the dependence of electrophoretic mobility on the pertinent parameters is made in Section 4 followed by a brief summary of all the results in Section 5.

## 2 MATHEMATICAL MODEL

This article deals with the electrophoresis of a soft particle comprised of charged and hydrophobic rigid inner core coated with ion and fluid permeable PEL. The surface charge density and hydrodynamic slippage of the inner core are denoted as σ and β, respectively. The particle immersed in binary valence symmetric z:z electrolyte with bulk concentration $n_{0}$ (in mM). We consider the typical situation in which the particle radius is substantially greater than the EDL thickness. For such a case, we may use flat‐plate theory [17, 18, 19, 20, 21, 22, 23] to investigate the undertaken problem. It should be emphasized that under this assumption, we may simply ignore the particle's curvature effect, and so the applied electric field can be regarded parallel to the particle surface. It is worth noting that the deduced results for electrophoretic mobility from the flat‐plate formalism is applicable not only to a strictly planner surface, but also to a big spherical soft particle with a thin double layer limit for which the electric field is parallel to the particle surface. Figure S.1 presented in the Supporting Information schematically depicts our current problem under flat‐plate representation.

The strength of the applied electric field is considered to be weak enough so that the electrophoretic velocity varies linearly with the electric field. Thus, we may write electrophoretic velocity as $U_{E}=\mu_{E}E$, where $\mu_{E}$ is the electrophoretic mobility and E refers to the strength of the electric field. Note that the moderate to high strength of applied electric field often induces the unwanted Joule heating effect. In this work, we used the exponential distribution [47, 48] to account for the distribution of monomers and accompanied volume charge distributed within the PEL, which is defined as

(1) $$h\left(x\right)=\exp\left(-\frac{x}{d}\right):0\leq x<\infty ,$$

where the maximum segment density and consequently the thickness of PEL is represented by d. Within the PEL, the presence of an ionizable functional group results in a nonzero volumetric charge. Since the monomers across the PEL are distributed inhomogeneously, they thus lead a similar spatial dependence of the PEL‐charge. Thus, the local volume charge density of the PEL is given by [48]

(2) $$\rho_{fix}=\rho_{0}h\left(x\right).$$

Here, $\rho_{0}=ZFN$ denotes the nominal volume charge density within the PEL, where F, Z, and N refer to the Faraday constant, valence, and molar concentration of the functional group distributed within PEL, respectively.

In order to model the electrokinetic transport phenomena, one often considers the Boltzmann distribution for spatial distribution of mobile electrolyte ions present in the aquatic microenvironment. Note that Boltzmann distribution in general consider the ions as point‐like charge and neglect the interaction among finite sized electrolyte ions. Such a consideration may lead to a sufficiently large accumulation of counterions near a moderate to highly charged surface. Thus, for electrophoresis of moderate to highly charged particle, the ion steric effect comes into play and size of mobile electrolyte ions needs to incorporate in their spatial distribution. Based on the Carnahan–Striling model [36, 37, 38], the modified Boltzmann distribution, which accounts for the interaction among finite‐sized ions is given as

(3) $$n_{i}\left(x\right)=\frac{\gamma^{\infty }}{\gamma (x)}n_{0}\exp\left(-\frac{z_{i}e\psi \left(x\right)}{k_{B}T}\right),$$

where ψ(x), $k_{B}$, T, and e refer to the EDL potential, Boltzmann constant, absolute temperature, and elementary charge, respectively. The ion activity coefficient γ(x) appearing in Equation (3) is given as follows:

(4) $$\gamma \left(x\right)=\exp\left[\frac{\phi \left(x\right)\{8-9\phi \left(x\right)+3\phi^{2}\left(x\right)\}}{{\left\{1-\phi \left(x\right)\right\}}^{3}}\right].$$

The quantity $\gamma^{\infty }$ represents the bulk values of γ(x) (i.e., $\gamma \left(x\right)\to \gamma^{\infty }$ as x→∞), and is defined by

(5) $$\gamma^{\infty }=\exp\left[\frac{\phi_{B}\left(8-9\phi_{B}+3\phi_{B}^{2}\right)}{{\left(1-\phi_{B}\right)}^{3}}\right].$$

The total ion volume fraction at a location x is represented as $\phi \left(x\right)=\phi_{B}\left(n_{1}\left(x\right)+n_{2}\left(x\right)\right)/2n_{0}$, where $\phi_{B}$ indicates the total volume fraction of bulk electrolyte, defined as $\phi_{B}=\left(4\pi r_{i}^{3}/3\right)2N_{A}n_{0}$. The Avogadro number is denoted by $N_{A}$, and the radius of mobile electrolyte ions is $r_{i}$ (i=1,2). We consider the typical situation where the radius of cation and anions are same [49], that is, $r_{1}=r_{2}=r$ say, and for such a case, the term $\gamma^{\infty }/\gamma \left(x\right)$ may be approximated as follows [50]:

(6) $$\frac{\gamma^{\infty }}{\gamma (x)}=\frac{1}{1-P\left\{1-\cosh\left(\frac{ze\psi (x)}{k_{B}T}\right)\right\}},$$

where $P=8\phi_{B}/\left(1+8\phi_{B}\right)$ refers to the steric factor, which accounts for the impact of ion steric effect. Taking ion size into consideration, the Poisson–Boltzmann equation for electrostatic potential distribution may be written as

(7) $$\frac{d^{2}\psi }{dx^{2}}=-\frac{\rho_{el}\left(x\right)+\rho_{fix}\left(x\right)}{\varepsilon_{e}},\;\;0\leq x<\infty ,$$

where $\varepsilon_{e}$ represents the dielectric permittivity of the electrolyte medium. The term $\rho_{el}\left(x\right)$ denotes the volumetric charge due to mobile ions in the electrolyte solution, given as

(8) $$\rho_{el}\left(x\right)=\sum_{i=1}^{2}z_{i}Fn_{i}\left(x\right).$$

Substituting (3) into (7), we may deduce the governing equation for EDL potential, given by

(9) d2ψ(x)dx2=−1εe−2zFn0sinhzeψ(x)kBT1−P1−coshzeψ(x)kBT+ρ0exp−xdsinhzeψ(x)kBT1−P1−coshzeψ(x)kBT,0≤x<∞.

The inner core of the undertaken particle bears uniform surface charge density σ. Besides, the electric potential and its gradient vanish along the far field. Thus, the boundary conditions associated to the EDL potential may be given as

(10a) $$\begin{array}{cc} & \frac{d\psi }{dx}{|}_{x=0}=-\frac{\sigma }{\varepsilon_{e}}, \end{array}$$

(10b) $$\begin{array}{cc} & \psi \left(x\right){|}_{x\to \infty }=0,\;\;\text{and}\;\;\frac{d\psi }{dx}{|}_{x\to \infty }=0. \end{array}$$

Note that, for the present description of monomer as well as the accompanying charge, distribution across the PEL gradually approaches to zero. Thus, there is no need to specify boundary conditions for EDL potential as well as any flow variables across such a PEL‐to‐electrolyte interface. The governing equation for EDL potential as well as associated boundary conditions are rewritten in scaled form as follows:

(11) $$\begin{array}{ccc} \frac{d^{2}y}{d{\bar{x}}^{2}} & = & {\left(\kappa d\right)}^{2}\left\{\frac{\sinh y}{1+2P\sinh^{2}\left(\frac{y}{2}\right)}-\frac{ZFN}{2zen_{0}}\exp\left(-\bar{x}\right)\right\},\\ & & \;0\leq \bar{x}<\infty \end{array}$$

subject to

(12a) $$\begin{array}{cc} & \frac{dy}{d\bar{x}}=-\bar{\sigma ,} \end{array}$$

(12b) $$\begin{array}{cc} & y\left(\bar{x}\right)\to 0\;\;\;\text{as}\;\;\;\bar{x}\to \infty . \end{array}$$

Here, $\kappa =\sqrt{2z^{2}eFn_{0}/\varepsilon_{e}k_{B}T}$ defines the inverse of the EDL thickness. The nondimensional quantities are $\bar{x}=x/d$, $y\left(\bar{x}\right)=\psi \left(x\right)/\phi_{0}$, where, $\phi_{0}=k_{B}T/ze$. The term $\bar{\sigma }=\sigma d/\varepsilon_{e}\phi_{0}$ refers to the scaled surface charge density of the inner core.

The exponential distribution of monomers across the PEL leads to the spatially varying frictional coefficient τ(x), defined as [47, 48]

(13) $$\tau \left(x\right)=\tau_{0}h\left(x\right),\;\;\;0\leq x<\infty ,$$

where $\tau_{0}$ represents the nominal frictional coefficient of the PEL. The motion of an ionized, incompressible fluid within PEL with a spatially dependent friction coefficient is governed by

(14) $$\eta \frac{d^{2}u}{dx^{2}}-\tau_{0}h\left(x\right)u\left(x\right)+\rho_{el}\left(x\right)E=0,\;\;\;0\leq x<\infty ,$$

where the fluid viscosity is denoted as η. The appropriate boundary conditions associated with the fluid flow equation (14) are given below

(15a) $$\begin{array}{cc} & u\left(x\right){|}_{x=0}=\beta \frac{du}{dx}{|}_{x=0}, \end{array}$$

(15b) $$\begin{array}{cc} & u\left(x\right)\to -U_{E}\;\;\;\text{as}\;\;\;x\to \infty . \end{array}$$

The condition (15a) refers to the Navier‐slip velocity boundary condition, which relates to the velocity along the surface of the inner core with the velocity gradient thereat. The hydrodynamic slip length is represented by the proportionality constant β appearing in Equation (15a). The boundary condition given in (15b) refers the far‐field fluid velocity. In order to solve the fluid flow equation (14) subject to the given condition (15), we introduce a change in variable, defined as

(16) $$t=2\lambda d\exp\left(-\frac{x}{2d}\right),$$

where the Brinkman screening length is denoted as $\lambda^{-1}$ (=$\sqrt{\eta /\tau }$), which defines the flow permeability across the PEL. Higher the value of $\lambda^{-1}$, the strength of electroosmotic flow (EOF) across the PEL further increases. Using the above transformation, we have solved Equation (14) subject to the boundary conditions ((15a), (15b)), and the velocity field in the transformed coordinate system may be deduced as follows:

(17) $$\begin{array}{ccc} u(t) & = & I_{0}\left(t\right)\int_{0}^{t}tK_{0}\left(t\right)G\left(t\right)Edt-K_{0}\left(t\right)\int_{0}^{t}tG\left(t\right)I_{0}\left(t\right)Edt\\ & & -I_{0}\left(t\right)\left[\int_{0}^{2\lambda d}\;tK_{0}\left(t\right)G\left(t\right)Edt-\frac{K_{0}\left(2\lambda d\right)-\beta \lambda K_{1}\left(2\lambda d\right)}{I_{0}\left(2\lambda d\right)+\beta \lambda I_{1}\left(2\lambda d\right)}\right.\;\;\;\;\\ & & \left.\;\int_{0}^{2\lambda d}tI_{0}\left(t\right)G\left(t\right)Edt\right], \end{array}$$

where $I_{n}\left(t\right)$ and $K_{n}\left(t\right)$ are the modified nth‐order Bessel functions of 1st and 2nd kind, respectively. The expression (17) involves the function G(t), defined as

(18) $$G\left(t\right)=-\rho_{el}\left(x\right)\left(\frac{4d^{2}}{\eta t^{2}}\right).$$

## 3 ELECTROPHORETIC MOBILITY

From the relation (15b) and (16), the electrophoretic mobility may be obtained from the following relation:

(19) $$\mu_{E}=-u\left(x\to \infty \right)/E=u\left(t\to 0\right)/E.$$

Using the velocity field given in (17) and above limit (19), the electrophoretic mobility of the particle may be deduced as follows:

(20) $$\begin{array}{ccc} \mu_{E} & = & \int_{0}^{2\lambda d}tK_{0}\left(t\right)G\left(t\right)dt\\ & & -\frac{K_{0}\left(2\lambda d\right)-\beta \lambda K_{1}\left(2\lambda d\right)}{I_{0}\left(2\lambda d\right)+\beta \lambda I_{1}\left(2\lambda d\right)}\int_{0}^{2\lambda d}tI_{0}\left(t\right)G\left(t\right)dt.\; \end{array}$$

We adopt suitable numerical technique to evaluate the electrophoretic mobility from the above relation. The detailed description of the numerical method is summarized in Supporting Information S.2. In addition to the numerical results, we further calculate the analytical results for the electrophoretic mobility of the undertaken particle under various limiting situations, which are presented subsequently. The validation of numerical results is further indicated in Supporting Information S.5.

### 3.1 Theoretical derivation of electrophoretic mobility under limiting cases

#### 3.1.1 Electrophoretic mobility for weakly charged particle

Within in the Debye–Hückel electrostatic framework valid for weakly charged particle, we deduce the analytical results for electrophoretic mobility. The detailed step to calculate the analytical results for electrophoretic mobility is summarized in Supporting Information S.3. In order to highlight the contribution of PEL‐charge and surface charge of the inner core, we write the electrophoretic mobility as $\mu_{E}=\mu_{P}+\mu_{S}$, where $\mu_{P}$ and $\mu_{S}$ refer to the contribution from PEL‐charge and surface charge of the inner core, respectively. After performing algebraic simplification, we may deduce the explicit form of $\mu_{P}$ and $\mu_{S}$, as follows

(21) $$\begin{array}{ccc} \mu_{P} & = & \frac{\rho_{0}}{\eta \lambda^{2}}\left[\frac{{\left(\kappa d\right)}^{2}}{{\left(\kappa d\right)}^{2}-1}\{1-2\lambda dBI_{1}\left(2\lambda d\right)-2\lambda dK_{1}\left(2\lambda d\right)\}\right.\\ & & +\frac{{\left(\lambda d\right)}^{2}}{{\left(\kappa d\right)}^{2}-1}\{2\left(\gamma +B+\ln\left(\lambda d\right)\right)\kappa d\sum_{n=0}^{\infty }\frac{{\left(\lambda d\right)}^{2n}}{{\left(n!\right)}^{2}\left(\kappa d+n\right)}\\ & & -\kappa d\sum_{n=0}^{\infty }\frac{{\left(\lambda d\right)}^{2n}}{{\left(n!\right)}^{2}{\left(\kappa d+n\right)}^{2}}\\ & & \left.-2\kappa d\sum_{n=0}^{\infty }\frac{{\left(\lambda d\right)}^{2n}}{{\left(n!\right)}^{2}\left(\kappa d+n\right)}\left(\sum_{k=1}^{n}\frac{1}{k}\right)\}\right] \end{array}$$

and

(22) $$\begin{array}{ccc} \mu_{S} & = & \frac{\sigma }{\eta \kappa }{\left(\kappa d\right)}^{2}\left[\sum_{n=0}^{\infty }\frac{{\left(\lambda d\right)}^{2n}}{{\left(n!\right)}^{2}{\left(\kappa d+n\right)}^{2}}\right.\\ & & -2\left\{\gamma +B+\ln(\lambda d)\right\}\sum_{n=0}^{\infty }\frac{{\left(\lambda d\right)}^{2n}}{{\left(n!\right)}^{2}\left(\kappa d+n\right)}\;\\ & & \left.+2\sum_{n=0}^{\infty }\frac{{\left(\lambda d\right)}^{2n}}{{\left(n!\right)}^{2}\left(\kappa d+n\right)}\left(\sum_{k=1}^{n}\frac{1}{k}\right)\right]. \end{array}$$

The above expressions for mobility involve the term B, defined as

(23) $$B=\frac{K_{0}\left(2\lambda d\right)-\beta \lambda K_{1}\left(2\lambda d\right)}{I_{0}\left(2\lambda d\right)+\beta \lambda I_{1}\left(2\lambda d\right)}$$

and γ is Euler's constant with γ=0.577···. Note that the expression (21) for the contribution of mobility from PEL‐charge has a singularity at κd=1. For such a special case, the expression for G(t) may be deduced as

(24) $$G\left(t\right)=\frac{\rho_{0}}{2\eta \lambda^{2}}\left\{1-2\ln\left(\frac{t}{2\lambda d}\right)\right\}.$$

Substituting the above relation (24) into (20), we may deduce the contribution of electrophoretic mobility due to PEL‐charge valid for κd=1 as follows:

(25) μP=ρ0ηλ212+γ+ln(λd)+K0(2λd)−λdK1(2λd)+B{1−I0(2λd)−λdI1(2λd)}12.

We further consider the typical situation for which the inner core is hydrophilic in nature, that is, β=0. Under such a limit, the contribution of electrophoretic mobility from the PEL‐charge and charge properties of the inner core are as follows:

(26) $$\begin{array}{ccc} \mu_{P} & = & \frac{\rho_{0}}{\eta \lambda^{2}}\left[\frac{{\left(\kappa d\right)}^{2}}{{\left(\kappa d\right)}^{2}-1}\{1-\frac{1}{I_{0}\left(2\lambda d\right)}\}+\frac{{\left(\lambda d\right)}^{2}}{{\left(\kappa d\right)}^{2}-1}\right.\\ & & \times \{2\left(\gamma +\frac{K_{0}\left(2\lambda d\right)}{I_{0}\left(2\lambda d\right)}+\ln\left(\lambda d\right)\right)\kappa d\sum_{n=0}^{\infty }\frac{{\left(\lambda d\right)}^{2n}}{{\left(n!\right)}^{2}\left(\kappa d+n\right)}\\ & & -\kappa d\sum_{n=0}^{\infty }\frac{{\left(\lambda d\right)}^{2n}}{{\left(n!\right)}^{2}{\left(\kappa d+n\right)}^{2}}\\ & & \left.-2\kappa d\sum_{n=0}^{\infty }\frac{{\left(\lambda d\right)}^{2n}}{{\left(n!\right)}^{2}\left(\kappa d+n\right)}\left(\sum_{k=1}^{n}\frac{1}{k}\right)\}\right] \end{array}$$

and

(27) $$\begin{array}{ccc} \mu_{S} & = & \frac{\sigma }{\eta \kappa }{\left(\kappa d\right)}^{2}\left[\sum_{n=0}^{\infty }\frac{{\left(\lambda d\right)}^{2n}}{{\left(n!\right)}^{2}{\left(\kappa d+n\right)}^{2}}\right.\\ & & -2\left\{\gamma +\frac{K_{0}\left(2\lambda d\right)}{I_{0}\left(2\lambda d\right)}+\ln\left(\lambda d\right)\right\}\sum_{n=0}^{\infty }\frac{{\left(\lambda d\right)}^{2n}}{{\left(n!\right)}^{2}\left(\kappa d+n\right)}\\ & & \left.+2\sum_{n=0}^{\infty }\frac{{\left(\lambda d\right)}^{2n}}{{\left(n!\right)}^{2}\left(\kappa d+n\right)}\left(\sum_{k=1}^{n}\frac{1}{k}\right)\right]. \end{array}$$

The above expressions agree well with the results deduced by [48] (see Equations (21) and (22)). For the sake of convenience, we have recast the same below:

(28) μP=ρ0ηλ2(κd)2(κd)2−1∑n=0∞1−1I0(2λd)−1I0(2λd)[Γ(κd)]2κd∑n=0∞(λd)2n+2[Γ(κd+n+1)]2

and

(29) $$\mu_{S}=\frac{\sigma_{0}}{\eta \kappa }\frac{{\left[\Gamma \left(\kappa d+1\right)\right]}^{2}}{I_{0}\left(2\lambda d\right)}\sum_{n=0}^{\infty }\frac{{\left(\lambda d\right)}^{2n}}{{\left[\Gamma \left(\kappa d+n+1\right)\right]}^{2}}.$$

Note that the expressions (26) and (27), respectively, take different forms compared to corresponding expressions (28) and (29), however, they agree numerically with each other.

It is interesting to note that the shielding effect increases with the rise in κd. For sufficiently large κd (i.e., κd>>1), the counterpart of mobility $\mu_{S}$ induced due to the charge density of the inner core vanishes due to a sufficiently high impact of shielding effect [24, 25]. On the contrary, the counterpart of electrophoretic mobility induced due to nonzero PEL‐charge is nonzero, which is the main characteristic feature of the electrokinetics soft particles [24, 25, 28]. Thus, for the undertaken particle, under κd>>1 limit, $\mu_{S}=0$ and the counterpart of electrophoretic mobility induced due to weakly PEL‐charge reduced to the following expression:

(30) $$\mu_{P}=\frac{\rho_{0}}{\eta \lambda^{2}}\left[1-2\lambda dBI_{1}\left(2\lambda d\right)-2\lambda dK_{1}\left(2\lambda d\right)\right].$$

If we further consider λd≫1 counterpart of electrophoretic mobility induced due to weakly PEL‐charge reduced to

(31) $$\mu_{P}⟶\frac{\rho_{0}}{\eta \lambda^{2}}.$$

Note that λd>>1 refers to the typical situation for which the Brinkmann screening length of the PEL is sufficiently small. Thus, the flow penetration across the PEL is negligible, which in turn leads to the mobility expression (31) as independent of slip length, and approaches to that of the soft particle with a hydrophilic inner core [28]. We further consider another extreme situation where κd<<1. Under such a limit, the contribution of electrophoretic mobility arising from PEL‐charge and charge density of the inner core reduces to the following limits:

(32) $$\mu_{P}=\frac{\rho_{0}d}{\eta \kappa }\left[1-2\kappa d\left\{\gamma +B+\ln(\lambda d)\right\}\right],$$

(33) $$\mu_{S}=\frac{\sigma }{\eta \kappa }\left[1-2\kappa d\left\{\gamma +B+\ln(\lambda d)\right\}\right].$$

#### 3.1.2 Approximate mobility expression valid for arbitrarily charged particle with smaller range in κd

We have further deduced an approximate analytical form of electrophoretic mobility valid for arbitrarily charged soft particle and smaller range in κd. The detailed step to deduce the following analytical expression is summarized in Supporting Information S.4. Under the said limit, the mobility expression is as follows:

(34) μE=ρ0ηλ2A(κd)2A(κd)2−1{1−2λdK1(2λd)−B2λdI1(2λd)}∑k=1n1k+A(λd)2A(κd)2−1{2(γ+B+ln(λd))κd×∑n=0∞(λd)2n(n!)2(Aκd+n)−κd∑n=0∞(λd)2n(n!)2(Aκd+n)2−2κd∑n=0∞(λd)2n(n!)2(Aκd+n)∑k=1n1k}+ψ0εeηA(κd)2∑n=0∞(λd)2n(n!)2(Aκd+n)2−2{γ+B+lnλd}∑n=0∞(λd)2n(n!)2(Aκd+n)+2∑n=0∞(λd)2n(n!)2(Aκd+n)∑k=1n1k.

## 4 RESULTS AND DISCUSSION

We have now presented some of the representative results in this section. It is important to note that the particle mobility depends on several intrinsic parameters, including charge property (e.g., polarity and molar concentration of PEL‐charge), thickness, and Brinkman screening length of PEL; charge density and hydrodynamic slip length of the inner core; concentration of bulk aqueous medium; size of ions, and so forth. The PEL is considered as either uncharged or charged with positive or negative polarity. We present the results considering surface charge density of the inner core is varied up to ± 80 ${\text{mC/m}}^{2}$ so that ion steric effect comes into play. Note that the ion steric effect has a substantial role when the electrostatic potential lies beyond the Debye–Hückel limit. The undertaken range for surface charge density of inner core of bioparticles is however reasonable [51]. The molar concentration of the functional group distributed within the PEL as well as electrolyte concentration is varied from low to high. We present the results considering various sizes of electrolyte ions. Note thatsynthesized colloidal entities coated with hydrophobic octadecyltrichlorosilane may yield a slip length of approximately 1 μm [52]. The hydrodynamic slip length is thus varied up to the order of micrometer. Note that rise in Brinkman screening length increases the flow permeability of fluid across the PEL. In general, for soft biocolloids, the softness parameter λd associated to Brinkmann screening length can range up to 100 [53, 54]. Below we have indicated the dependence of electrophoretic mobility on the aforementioned pertinent parameters. We present the results for scaled electrophoretic mobility $\mu_{E}/\mu_{0}$, scaled by $\mu_{0}=\varepsilon_{e}\phi_{0}/\eta$.

**FIGURE 1:**
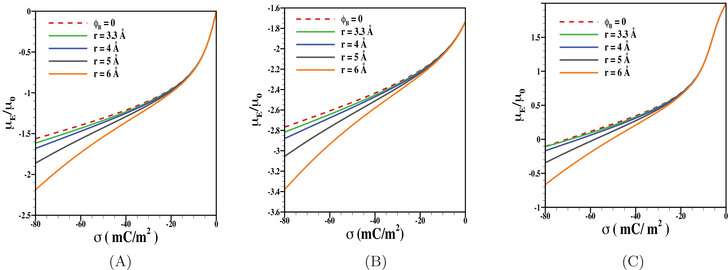
Scaled electrophoretic mobility $\mu_{E}/\mu_{0}$ is shown as a function of charge density of the inner core σ for various values of r (= 3.3 Å, 4 Å, 5 Å, 6 Å) and $\phi_{B}=0$ with fixed values of (A) N=0, (B) Z=−1, N=10 mM, and (C) Z=1, N=10 mM. The results are shown for the fixed values of d=10 nm, β=1 nm, $\lambda^{-1}=5$ nm, and electrolyte concentration $n_{0}=1$ mM.

We have first shown the results for electrophoretic mobility as a function of charge density of the inner core considering fixed values of d=10 nm, β=1 nm, and $\lambda^{-1}=5$ nm (Figure 1). The results are presented here considering PEL as uncharged (Figure 1A) or charged with negative (Figure 1B) as well as positive (Figure 1C) polarity. In order to highlight the impact of ion steric effect, we have presented the results considering electrolyte ions as point‐like charges (e.g., $\phi_{B}=0$) or having finite size with r=3.3,4,5, and 6 Å, respectively. The consideration of such sizes of mobile ions is rather common [55]. As expected, the particle mobility is negative for negatively charged inner core coated with either uncharged PEL or negatively charged PEL (Figure 1A,B). On the other hand, we observe a mobility reversal when the particle consists of a negatively charged core coated with positively charged PEL (Figure 1C). For all three cases presented in Figure 1, we observe that the ion size effect has a substantial role on the particle mobility and their impact is more pronounced when the net particle charge (PEL as well as inner core) is moderate to high. Note that the finite size of ions prevents the accumulation of sufficiently high number of counterions near a moderate to highly charged surface as well as within the PEL, which in turn reduces the impact of shielding effect compared to that for point‐like ions. As a result, the net neutralization of particle charge due to finite sized electrolyte ions is less, which in turn leads to larger electrophoretic mobility compared to that for point‐like ions. With a similar analogy, the magnitude in electrophoretic mobility further increases with the rise in ion size due to further reduction in impact of the shielding effect.

**FIGURE 2:**
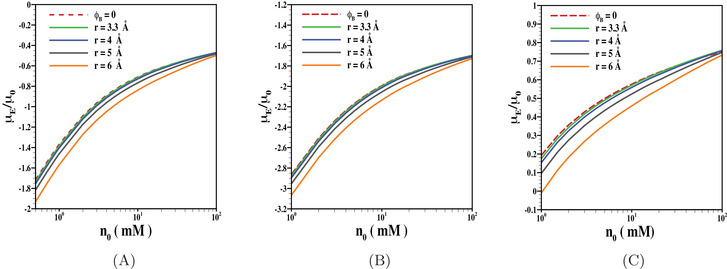
Scaled electrophoretic mobility $\mu_{E}/\mu_{0}$ is shown as a function of $n_{0}$ for various values of r (= 3.3 Å, 4 Å, 5 Å, 6 Å) and $\phi_{B}=0$ with fixed values of (A) N=0, (B) Z=−1, N=10 mM, and (C) Z=1, N=10 mM. The results are shown for the fixed values of, σ=−50 ${\text{mC/m}}^{2}$, d=10 nm, β=1 nm, $\lambda^{-1}=5$ nm.

In Figure 2, we have shown the electrophoretic mobility as a function of electrolyte concentration for various choices of ion size. The results are presented for uncharged (Figure 2A,B), negatively charged (Figure 2B), as well as positively charged (Figure 2C) PEL considering fixed values of other pertinent parameters, which are further summarized in the caption of the said figure. It may be noted that the impact of volume fraction, which depends on ion size and electrolyte concentration, has a substantial impact on the particle mobility. Note that for a smaller range of electrolyte concentration, the neutralization of core as well as PEL‐charge is minimum due to less impact of shielding effect arising from accumulation of counterions near the surface of inner core and within the PEL. However, the impact of the shielding effect grows with the rise in electrolyte concentration and thus, leads to a reduction in net particle charge. As a result, the impact of ion size on the particle motion reduces gradually. For uncharged PEL or similarly charged inner core and PEL, the enhanced shielding effect with the rise in electrolyte concentration leads to a reduction in magnitude in particle mobility (Figure 2A,B). The situation is further complicated for the case when the core and PEL are oppositely charged. For the undertaken parameters considered in Figure 2C, we observed that the particle mobility is positive for the entire range in electrolyte concentration and the mobility is further augmented with the rise in electrolyte concentration. Note that for a smaller range in electrolyte concentration, the impact of inner core charge is significant, which further reduces the impact of PEL‐charge. On the other hand, with the rise in electrolyte concentration, the impact of positively charged PEL overwhelms the effect of negatively charged inner core. The competition of impact of oppositely charged inner core and PEL defines the particle mobility.

**FIGURE 3:**
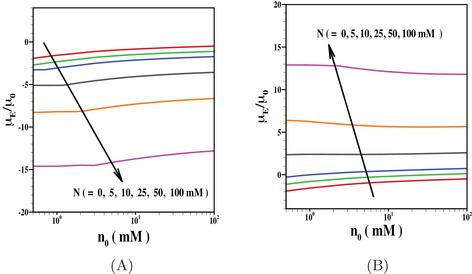
Scaled electrophoretic mobility $\mu_{E}/\mu_{0}$ is shown as a function of $n_{0}$ for various values of N (= 0, 5 mM, 10 mM, 25 mM, 50 mM, 100 mM) with fixed values of (A) Z=−1 and (B) Z=1. The results are shown for the fixed values of σ=−50 ${\text{mC/m}}^{2}$, r=6 Å, β=1 nm, $\lambda^{-1}=5$ nm.

From the earlier results presented in this section, it is evident that the inclusion of ion size effect provides more accurate results for particle mobility. We have now presented the results to indicate the dependence of particle mobility on the other pertinent parameters considering fixed value of ion size. In Figure 3, we have shown the impact of PEL‐charge on the electrophoretic mobility. The results are shown here as a function of electrolyte concentration considering the charged inner core is coated with either positively charged PEL (Figure 3B) or negatively charged PEL (Figure 3A). In both the results, we have further included the results for N=0 (uncharged PEL). The impact of ion steric effect and inhomogeneity is further considered in the results indicated in Figure 3 for fixed values of other model parameters. When the inner core and PEL are similarly charged, the PEL‐charge further strengthens the impact of the inner core, which in turn leads to an enhanced electrophoretic mobility. On the contrary, for negatively charged core coated with positively charged PEL, there is a competition of opposite charges residing in respective components. We observed a reversal in mobility for such a case. For both the cases, the magnitude of electrophoretic mobility however reduces with the rise in electrolyte concentration due to an enhanced shielding effect.

**FIGURE 4:**
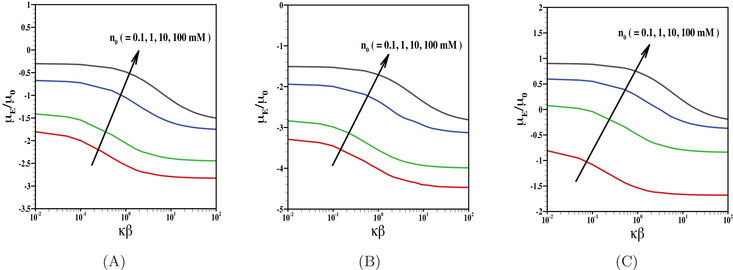
Scaled electrophoretic mobility $\mu_{E}/\mu_{0}$ is shown as a function of κβ for various values of $n_{0}$ (= 0.1 mM, 1 mM, 10 mM, 100 mM) with fixed values of (A) N=0, (B) Z=−1, N=10 mM, and (C) Z=1, N=10 mM. The results are shown for the fixed values of, σ=−50 ${\text{mC/m}}^{2}$, r=6 Å, d=10 nm, $\lambda^{-1}=5$ nm.

In Figure 4, we have presented the results for electrophoretic mobility as a function of κβ. The results are shown here for various choices of electrolyte concentration (or in other words, various choices of EDL thickness). We have shown the results for the particles with negatively charged inner core and is coated with uncharged (Figure 4A), negatively charged (Figure 4B) as well as positively charged PEL (Figure 4C) considering the effect of finite ion size with r=6Å. Note that the rise in κβ at a given EDL thickness increases the hydrodynamic slip length of the inner core. Rise in the hydrophobic behavior of the inner core has twofold effects on the particle motion, for example, reduction in hydrodynamic frictional force experienced by the particle as well as the reduction in charge neutralization of the inner core due to an enhanced EOF. Due to the combination of these two effects, the impact of the charge properties of the inner core is more pronounced for increasing the value of its hydrodynamic slippage. Thus, the particle comprised a negatively charged inner coated with uncharged (Figure 4A) or negatively charged PEL (Figure 4B), an enhanced mobility is achieved with the rise in hydrodynamic slippage. For the case when the PEL is oppositely charged to that of the inner core, a competition in charge properties of the core and shell layer occurs. Rise in electrolyte concentration of background aqueous medium leads to a reduction in Debye length, which in turn diminishes the contribution of the core charge density. As a result, with the rise in electrolyte concentration, the magnitude of particle mobility reduces for the case of uncharged PEL or negatively charged PEL. With a similar analogy, the reduced impact of the inner core leads to a positive mobility for the case when the PEL is positively charged. Thus, we can achieve a reversal in particle motion by suitably regulating the EDL thickness and hydrodynamic slippage.

**FIGURE 5:**
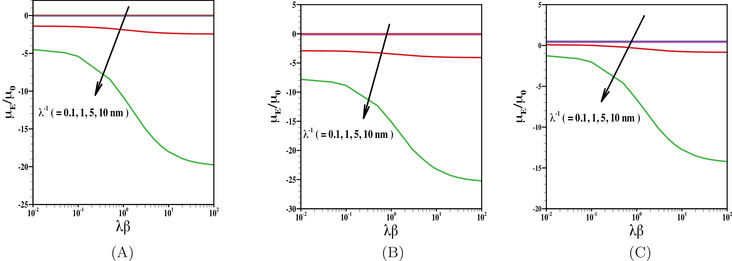
Scaled electrophoretic mobility $\mu_{E}/\mu_{0}$ is shown as a function of λβ for various values of $\lambda^{-1}$ (= 0.1 nm, 1 nm, 5 nm, 10 nm) with fixed values of (A) N=0, (B) Z=−1, N=10 mM, and (C) Z=1, N=10 mM. The results are shown for the fixed values of, σ=−50 ${\text{mC/m}}^{2}$, r=6 Å, d=10 nm, and molar concentration is 1 mM.

**FIGURE 6:**
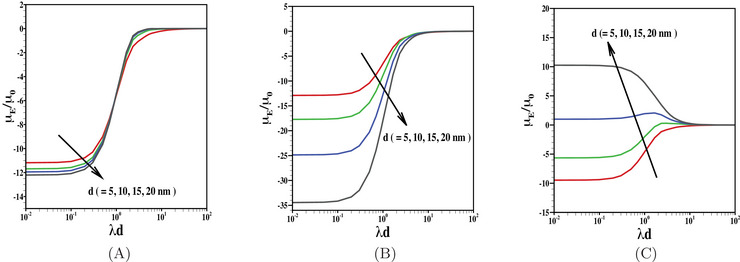
Scaled electrophoretic mobility $\mu_{E}/\mu_{0}$ is shown as a function of λd for various values of d (= 5 nm, 10 nm, 15 nm, 20 nm) with fixed values of (A) N=0, (B) Z=−1, N=10 mM, and (C) Z=1, N=10 mM. The results are shown for the fixed values of σ=−50 ${\text{mC/m}}^{2}$, r=6 Å, β=1 nm, and $n_{0}=1$ mM.

We further show the impact of hydrodynamic slippage of the inner core on the particle mobility by changing the Brinkmann screening length $\lambda^{-1}$ of the PEL. To illustrate the same, we have shown in Figure 5 the electrophoretic mobility as a function of λβ for four values of $\lambda^{-1}$. Note that rise in Brinkmann screening length increases the strength of EOF across the PEL. The enhanced EOF further reduces the counterion accumulation with the PEL and thereby reduces the neutralization of PEL‐charge, and thus enhances the electromotive force. As a result, the magnitude of particle mobility increases with the rise in $\lambda^{-1}$. We further observed that the impact of the hydrodynamic slippage of the inner core is more pronounced with the rise in $\lambda^{-1}$. On the contrary, smaller values of penetration length lead to an local increase in viscosity deep inside the PEL and thus, the flow of ambient fluid adheres near the outer edge of the PEL and leads to a reduced impact of hydrodynamic slippage of the inner core. The relative importance of PEL thickness as well as Brinkmann screening length on the particle motion is shown in Figure 6. The results are shown here for electrophoretic mobility as a function of λd for various choices of PEL thickness d. The choices of PEL thickness are however in line with the available studies on the electrophoresis of core‐shell structured soft particles [56, 57]. Note that a rise in d increases the PEL thickness as well as leads to an enhanced heterogeneity in monomer distribution across the PEL. Besides, for increasing d, the front edge of the PEL is more permeable to fluid flow and thus leads to a reduced counterion accumulation due to an enhanced impact of EOF of background electrolyte solution. Looking into the results presented in Figure 6A, we observed two different patterns in particle mobility with the rise in d. When PEL is highly permeable for a lower range of λd, the particle mobility even increases with the rise in d. For such a case, an augmented permeability of the PEL leads to such an enhanced mobility with the rise in d. On the other hand, when λd≥1, an increased d leads to a reduced mobility due to dense PEL. For such a case, the strength of EOF across the PEL reduces and thus, an enhanced counterion accumulation within the PEL may occur, which in turn reduces the net effective particle charge. In addition with the rise in λd, the frictional force experienced by the particle further enhances and thus, the particle mobility reduces (Figure 6A,B). As expected the particle switches its propulsion direction when the polarity of PEL‐charge is opposite to the surface charge of the inner core (Figure 6C). The impact of inner core charge is more pronounced and it overwhelms the PEL‐charge for the composite particle coated with thin PEL. On the contrary, the particle with thick PEL, the positively charged PEL dominates the particle motion and leads to positive electrophoretic mobility. In all the cases presented in Figure 6, it is evident that the particle mobility gradually approaches to zero for sufficiently high λd. Under such a limit, the net effective drag force experienced by the particle is sufficient enough to stop the particle motion.

## 5 CONCLUSIONS

An extensive mathematical study on the electrophoresis of core‐shell structured soft particles is made. The undertaken particle is composed of a charged and hydrophobic inner core and coated with an uncharged or charged absorbed polymer layer. The monomer as well as the charge distribution across the peripheral shell layer follow the exponential distribution. Within the Debye–Hückel electrostatic framework, we deduced closed form analytical results for particle mobility considering combined effects of hydrodynamic slippage and inhomogeneous charge distribution across the shell layer. Within the aforesaid limit, a number of analytical expressions are deduced for various electrohydrodynamic conditions. In addition, we further deduce an approximate analytical expression for electrophoretic mobility valid for arbitrarily charged particle with smaller range in κd. In addition to the analytical results, we further deduced numerical results for electrophoretic mobility without imposing any restriction on physical parameters associated to the undertaken problem. The results are presented considering the charged inner core coated with uncharged PEL, or the PEL and inner core may bear similar or dissimilar electrostatic charges. When the electrostatic charges residing along the surface of the inner core and within the shell PEL are of opposite polarity, the particle may switch its propulsion direction. For such a case, a competition of electrostatic charge of both the components determines the particle motion. We observed that the impact of shielding reduces with the rise in ion size, which leads to an enhanced electrophoretic mobility. The impact of heterogeneity in monomers and immobile charges resides in PEL as well as Brinkmann screening length has a substantial impact on the overall particle motion. The decrease in value of Brinkmann screening length leads to an enhanced neutralization of particle charge and leads to a decrease in particle mobility, which further approaches zero for sufficiently small values of screening length. The impact of hydrodynamic slippage enhances with the rise in slip length and its impact further pronounced for smaller thickness of EDL as well as highly permeable PEL.

## CONFLICT OF INTEREST STATEMENT

The authors have declared no conflict of interest.

## Supporting information

Supporting Information

## Data Availability

Data supporting the findings of this study are available from the corresponding author upon reasonable request.
