# Supplementary material for: Electrophoresis of polyelectrolyte‐adsorbed soft particle with hydrophobic inner core
Source: Electrophoresis. 2024 Sep 17;46(13-14):797–809. doi: 10.1002/elps.202400143 (PMC12449755; doi:10.1002/elps.202400143)
Supplement: Supplementary file 1 — Supporting Information [file ELPS-46--s001.pdf]

SUPPLEMENTARY MATERIAL

Electrophoresis of polyelectrolyte-adsorbed soft particle with hydrophobic inner core

Asim Mahata[a], S. K. Pal[a], H. Ohshima[b], Partha P. Gopmandal[c*]

[a] *Department of Mathematics, Jadavpur University Kolkata-70032, India*

[b] *Faculty of Pharmaceutical Sciences, Tokyo University of Science*
Noda, Chiba, Japan

[c] *Department of Mathematics, National Institute of Technology Durgapur*
Durgapur-713209, India

Nomenclature

d	Thickness of polyelectrolyte layer (PEL) (nm)
E	Applied electric field (V/m)
e	Elementary charge (C)
F	Faraday constant (C/mol)
k_B	Boltzmann constant (J/K)
N	Molar concentration fix ions distributed in PEL (mM)
n_0	Electrolyte concentration (mM)

*Corresponding author, e-mail: ppgopmandal.maths@nitdgp.ac.in, parthap1218@gmail.com, Telephone: +91-7250276690

N_A	Avogadro number (mol^{-1})
r	Radius of ion (Å)
T	Absolute temperature (K)
U_E	Electrophoretic velocity (m/s)
Z	Valence of the fix ions distributed in PEL
z	Valence of electrolyte ions

Greek symbols

β	Slip length (nm)
ϵ_e	Dielectric permittivity (F/m)
η	Fluid viscosity ($N{\cdot}s/m^2$)
$\gamma(x)$	Ion activity coefficient
γ	Euler's constant
κ	Inverse of the EDL thickness (nm)
λ^{-1}	Brinkmann screening length (nm)
μ_E	Electrophoretic mobility ($m^2V^{-1}s^{-1}$)
ϕ_B	Volume fraction
$\psi(x)$	EDL potential
ρ_{el}	Volumetric charge density of electrolyte ions
ρ_{fix}	Volumetric charge density of PEL
σ	Surface charge density (mC/m^2)
$\tau(x)$	Frictional coefficient

S.1 Schematic diagram

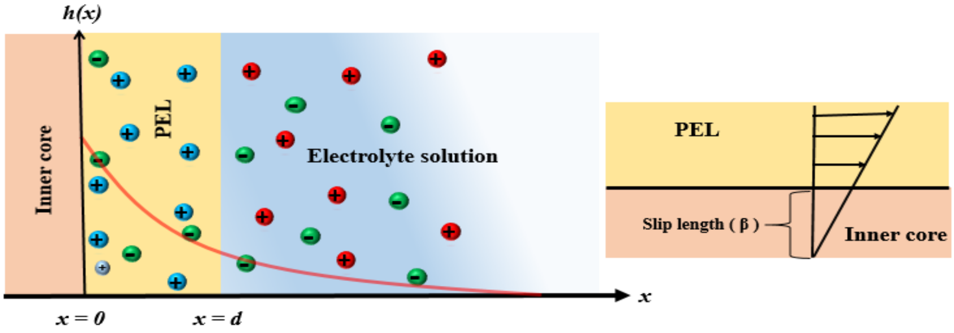

Figure S1: The schematic representation of electrophoresis of soft particle under flat-plate representation is shown. The schematic for variation of segment density as well as PEL-charge distribution is indicated. The slip length that characterizes the hydrodynamic slippage of the inner rigid core is further illustrated graphically.

S.2 Numerical method

Note that the relation (20) presented in main manuscript (MS) involves the function $G(t)$, which is defined in Eq. (18). The function $G(t)$ involves the net volume charge density ρ_{el}, which further involves the spatial distribution of mobile electrolyte ions. The electrostatic EDL potential and the spatial distribution of electrolyte ions are correlated via potential equation. Thus, in order to calculate the electrophoretic mobility, we first need the EDL potential. We adopt the finite difference based numerical scheme to discretize the governing equation for EDL potential given in equation Eq. (11) of main MS. The discretized equations are further solved by using tridiagonal matrix algorithm (TDMA) through an iterative manner. The iteration starts with an initial guess of EDL potential (say, zero potential) and continues till the absolute difference in two successive steps is smaller than the tolerance limit (say, 10^{-7}). With the converged results for EDL potential, we calculate the spatial distribution of electrolyte ions using the modified Boltzmann distribution given in relation Eq. (3) of main MS. Thus, with the known value of the spatial distribution of electrolyte ions, one can easily calculate net volume charge density ρ_{el} due to mobile electrolyte ions and hence, the function $G(t)$. Substituting $G(t)$ into Eq. (20) appearing in the main MS, and further performing the numerical integration we calculate the results for electrophoretic mobility. The validation of numerical scheme is shown in Section S.5.

S.3 Steps to calculate the electrophoretic mobility under Debye-Hückel limit

Within Debye-Hückel limit, we may recast the net charge density due to mobile electrolyte ions as follows

$$\rho_{el}(x) = -\varepsilon_e \kappa^2 \psi(x) \tag{S1}$$

Under this limit the EDL potential $\psi(x)$ may be deduced from linearized form of Eq. (9), with $P = 0$. After performing algebraic calculation, the EDL potential may be deduced as follows

$$\psi(x) = \frac{\rho_0}{\varepsilon_e \kappa^2}\left\{\frac{(\kappa d)^2 \exp\left(-\frac{x}{d}\right) - (\kappa d)\exp\left(-\kappa x\right)}{(\kappa d)^2 - 1}\right\} + \frac{\sigma}{\varepsilon_e \kappa}\exp\left(-\kappa x\right) \tag{S2}$$

Using the variable change as indicated in Eq. (16) of main MS, we may rewrite the electrostatic potential in terms of new variable t as follows

$$\psi(t) = \frac{\rho_0}{\varepsilon_e \kappa^2}\left\{\frac{\left(\frac{\kappa t}{2\lambda}\right)^2 - \kappa d\left(\frac{t}{2\lambda d}\right)^{2\kappa d}}{(\kappa d)^2 - 1}\right\} + \frac{\sigma}{\varepsilon_e \kappa}\left(\frac{t}{2\lambda d}\right)^{2\kappa d} \tag{S3}$$

Thus, we may deduce the close form expression for $G(t)$ given by

$$G(t) = \frac{\rho_0}{\eta\lambda^2}\left\{\frac{(\kappa d)^2}{(\kappa d)^2 - 1} - \frac{\kappa d}{((\kappa d)^2 - 1)}\frac{t^{2\kappa d - 2}}{(4\lambda^2 d^2)^{\kappa d - 1}}\right\} + \frac{\kappa\sigma}{\eta\lambda^2}\frac{t^{2\kappa d - 2}}{(4\lambda^2 d^2)^{\kappa d - 1}} \tag{S4}$$

Substituting Eq. (*S4*) into Eq.(20) of main MS, we may deduce the electrophoretic mobility as follows

$$\begin{aligned}\mu_E \;=\; & \int_0^{2\lambda d} K_0(t)\left[\frac{\rho_0}{\eta\lambda^2}\left\{\frac{(\kappa d)^2}{(\kappa d)^2 - 1}t - \frac{\kappa d}{((\kappa d)^2 - 1)}\frac{t^{2\kappa d - 1}}{(4\lambda^2 d^2)^{\kappa d - 1}}\right\} + \frac{\kappa\sigma}{\eta\lambda^2}\frac{t^{2\kappa d - 1}}{(4\lambda^2 d^2)^{\kappa d - 1}}\right]dt \\ & -\frac{K_0(2\lambda d) - \beta\lambda K_1(2\lambda d)}{I_0(2\lambda d) + \beta\lambda I_1(2\lambda d)}\int_0^{2\lambda d} I_0(t)\left[\frac{\rho_0}{\eta\lambda^2}\left\{\frac{(\kappa d)^2}{(\kappa d)^2 - 1}t - \frac{\kappa d}{((\kappa d)^2 - 1)}\frac{t^{2\kappa d - 1}}{(4\lambda^2 d^2)^{\kappa d - 1}}\right\} + \right. \\ & \left.\frac{\kappa\sigma}{\eta\lambda^2}\frac{t^{2\kappa d - 1}}{(4\lambda^2 d^2)^{\kappa d - 1}}\right]dt\end{aligned} \tag{S5}$$

Further substituting (S4) into (S5) and performing algebraic simplification we may deduce the analytical results for electrophoretic mobility. The explicit form of electrophoretic mobility is presented in main MS (please see eq. (21) and (22) appearing in main MS).

S.4 Steps to calculate the approximate mobility expression valid for arbitrarily charged particle with smaller range in κd

The modified Poisson-Boltzmann equation given in Eq. (9) of main MS may be rewritten as

$$\frac{d^2\psi(x)}{dx^2} = \kappa^2\phi_0 \left\{ \frac{\sinh\left(\frac{\psi(x)}{\phi_0}\right)}{1+\left(\frac{16\phi_B}{1+8\phi_B}\right)\sinh^2\left(\frac{\psi(x)}{2\phi_0}\right)} - \frac{\rho_0}{2zen_0}\exp\left(-\frac{x}{d}\right)\right\} \tag{S6}$$

We consider

$$\psi(x) = \psi^0(x) + \Delta\psi(x) \tag{S7}$$

where $\Delta\psi(x)$ is a small quantity and $\psi^0(x) = \psi(x=0)$. Eq. (*S7*) is a good approximation for small κx. We substitute Eq. (*S7*) into Eq. (*S6*) and linearize the Eq.(*S6*) with respect to $\Delta\psi(x)$ and thus

$$\frac{d^2\Delta\psi(x)}{dx^2} = \kappa^2\phi_0\left[A + A'\frac{\Delta\psi(x)}{\phi_0} - \frac{\rho_0}{2zen_0}\exp\left(-\frac{x}{d}\right)\right] \tag{S8}$$

where

$$A = \frac{\cosh\left(\frac{\psi^0(x)}{\phi_0}\right) + 8\phi_B}{(1+8\phi_B)\left\{1+\left(\frac{16\phi_B}{1+8\phi_B}\right)\sinh^2\left(\frac{\psi^0(x)}{2\phi_0}\right)\right\}^2} \tag{S9}$$

$$A' = \frac{\sinh\left(\frac{\psi^0(x)}{\phi_0}\right)}{1+\left(\frac{16\phi_B}{1+8\phi_B}\right)\sinh^2\left(\frac{\psi^0(x)}{2\phi_0}\right)} \tag{S10}$$

Equation (*S8*) need to be solve subject to the following boundary condition at the surface of the inner core with nonzero surface charge density σ, given by

$$\left.\frac{d\psi(x)}{dx}\right|_{x=0^+} = -\frac{\sigma}{\epsilon_e} \tag{S11}$$

Equation (*S8*) is solved and the explicit form of EDL potential may be deduced as follows

$$\psi(x) = \psi^0(x) - \frac{A'\phi_0}{A} + \frac{\rho_0 d}{\epsilon_e\kappa^2}\frac{\kappa d\exp\left(-\frac{x}{d}\right) - \frac{\exp\left(-\sqrt{A}\kappa x\right)}{\sqrt{A}}}{A(\kappa d)^2 - 1} + \frac{1}{\sqrt{A}\kappa}\frac{\sigma}{\epsilon_e}\exp\left(-\sqrt{A}\kappa x\right) \tag{S12}$$

By evaluating $\psi(x)$ given by Eq. (*S12*) at $x=0$, we obtain the following equation for $\psi^0(x) = \psi(0)$, given by

$$\frac{\sinh\left(\frac{\psi^0(x)}{\phi_0}\right)\sqrt{1+8\phi_B}}{\sqrt{\cosh\left(\frac{\psi^0(x)}{\phi_0}\right)+8\phi_B}} = \frac{ze\sigma}{\epsilon_e\kappa k_B T} \tag{S13}$$

Equation ($S12$) is a good approximation only for small κx, since Eq. ($S12$) is derived based on Eq.($S7$). For large κx, Eq. ($S12$) is no longer a good approximation. For large κx, $\psi(x)$ must becomes zero, that is,

$$\psi(x) \to 0 \qquad \text{as} \qquad \kappa x \to \infty \tag{S14}$$

However, Eq. ($S12$) tends to a non-zero limiting value, given by

$$\psi(x) \to \psi^0(x) - \frac{A'\phi_0}{A} \qquad as \qquad \kappa x \to \infty \tag{S15}$$

We thus introduce a convergence factor $\exp(-\alpha\kappa x)$ so that Eq. ($S12$) satisfies Eq. ($S14$), where α is an adjustable parameter. Thus, we may write

$$\begin{aligned}\psi(x) &= \left(\psi^0(x) - \frac{A'\phi_0}{A}\right)\exp(-\alpha\kappa x) + \frac{\rho_0 d}{\epsilon_e\kappa}\frac{\kappa d\exp\left(-\frac{x}{d}\right) - \frac{\exp(-\sqrt{A}\kappa x)}{\sqrt{A}}}{A(\kappa d)^2 - 1} + \\ &\quad \frac{1}{\sqrt{A}\kappa}\frac{ze\sigma}{\epsilon_e k_B T}\exp\left(-\sqrt{A}\kappa x\right)\end{aligned} \tag{S16}$$

If we choose $\alpha = \sqrt{A}$, we may deduce the following relation

$$\psi(x) = \psi^0(x)\exp\left(-\sqrt{A}\kappa x\right) + \frac{\rho_0 d}{\epsilon_e\kappa}\frac{\kappa d\exp\left(-\frac{x}{d}\right) - \frac{\exp(-\sqrt{A}\kappa x)}{\sqrt{A}}}{A(\kappa d)^2 - 1} \tag{S17}$$

where $\psi^0(x)$ is the solution to Eq. ($S13$). For the small $\psi^0(x)$ limit, we have that $A = 1$ and Eq. ($S17$) tends to the following correct limiting form, given by

$$\psi(x) = \frac{\sigma}{\epsilon_e\kappa}\exp(-\kappa x) + \frac{\rho_0 d}{\varepsilon_e\kappa}\frac{\kappa d\exp\left(-\frac{x}{d}\right) - \exp(-\kappa x)}{(\kappa d)^2 - 1} \tag{S18}$$

The velocity field governed by Eq. (14) of main MS can be rewritten as

$$\eta\frac{d^2u}{dx^2} - \tau h(x)u(x) = E\epsilon_e\left\{\frac{d^2\psi}{dx^2} + \frac{\rho_0}{\epsilon_e}\exp\left(-\frac{x}{d}\right)\right\}, \quad 0 \le x < \infty \tag{S19}$$

Using the relation Eq. (16) of main MS, the above equation transformed into the form

$$\frac{d^2u}{dt^2} + \frac{1}{t}\frac{du}{dt} - u(t) = \frac{E\epsilon_e}{\eta}\left\{\frac{d^2\psi}{dt^2} + \frac{1}{t}\frac{d\psi}{dt}\right\} + \frac{E\rho_0}{\eta\lambda^2} \tag{S20}$$

Using the transformed boundary conditions for flow field, the velocity field may be deduced as follows

$$\begin{aligned}u(t) &= \frac{E\epsilon_e}{\eta}\left[I_0(t)\int_0^t K_0(t)\left\{t\frac{d^2\psi}{dt^2} + \frac{d\psi}{dt}\right\}dt - K_0(t)\int_0^t I_0(t)\left\{t\frac{d^2\psi}{dt^2} + \frac{d\psi}{dt}\right\}dt\right] \\ &\quad + \frac{E\rho_0}{\eta\lambda^2}\left[I_0(t)\int_0^t tK_0(t)dt - K_0(t)\int_0^t tI_0(t)dt\right] \\ &\quad + I_0(t)\left[\frac{E\epsilon_e}{\eta}\left\{-\int_0^{2\lambda d} K_0(t)\left(t\frac{d^2\psi}{dt^2} + \frac{d\psi}{dt}\right)dt + B\int_0^{2\lambda d} I_0(t)\left(t\frac{d^2\psi}{dt^2} + \frac{d\psi}{dt}\right)dt\right\}\right. \\ &\quad \left. + \frac{E\rho_0}{\eta\lambda^2}\left\{-K_0(t)\int_0^{2\lambda d} tK_0(t)dt + B\int_0^{2\lambda d} tI_0(t)dt\right\}\right]\end{aligned} \tag{S21}$$

Using this expression and the relation (19) given in main MS, we may deduce the explicit form of electrophoretic mobility. The same is provided in eq. (35) of main MS.

S.5 Validation of numerical scheme

In this section, we have presented the validation of the numerical scheme adopted in the present study. We have shown the numerical results are in close agreement with the deduced analytical results under the Debye-Hückel limit valid for weakly charged particles, as well as an approximate analytical result for electrophoretic mobility valid for arbitrarily charged particles and lower range in κd. The validation of numerical results are shown subsequently.

S.5.1 Validation with the results deduced under Debye-Hückel limit

In Fig. S2, we have shown both the theoretical and numerical results for scaled electrophoretic mobility as a function of κd for various values of d. The results are presented considering the particle core is hydrophobic weakly charged and is coated with an uncharged PEL (Fig. S2(a)), negatively charged PEL (Fig. S2(b)), and positively charged PEL (Fig. S2(c)). The model parameters are chosen in such a manner that the Debye-Hückel approximation holds good. We observe a close agreement of the numerical results with the deduced theoretical results under a low charge limit.

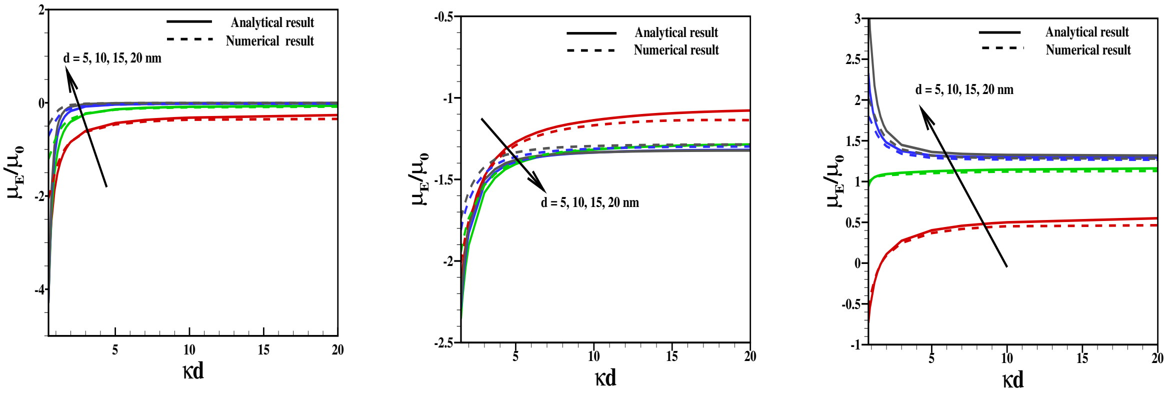

(a) (b) (c)

Figure S2: Scaled electrophoretic mobility μ_E/μ_0, scaled by $\mu_0 = (\epsilon_e \phi_0)/\eta$ is shown as a function of κd for various values of d (= 5 nm, 10 nm, 15 nm, 20 nm) with fixed values of (a) $N = 0$, (b) $Z = -1$, $N = 10$ mM, and (c) $Z = 1$, $N = 10$ mM. The results are shown for the fixed values of $\sigma = -10$ mC/m^2, $r = 6$ Å, $\beta = 1$ nm. Solid lines refers the deduced analytical results and dashed lines refers the numerical results for scaled electrophoretic mobility.

S.5.2 Validation with approximate results for electrostatic potential and electrophoretic mobility

In Fig. S3, we have shown the results for the spatial distribution of scaled electrostatic potential considering uncharged PEL (Fig. S3(a)), negatively charged PEL (Fig. S3(b)) and positively charged PEL (Fig. S3(c)). The results are shown here for $\kappa d = 0.9, 1, 5$ and 10. Here we present the deduced numerical results for EDL potential as well as approximate analytical results for EDL potential as indicated in Eq. (S17). As expected the numerical results are in a close agreement with the approximate analytical results for EDL potential for smaller range in κd.

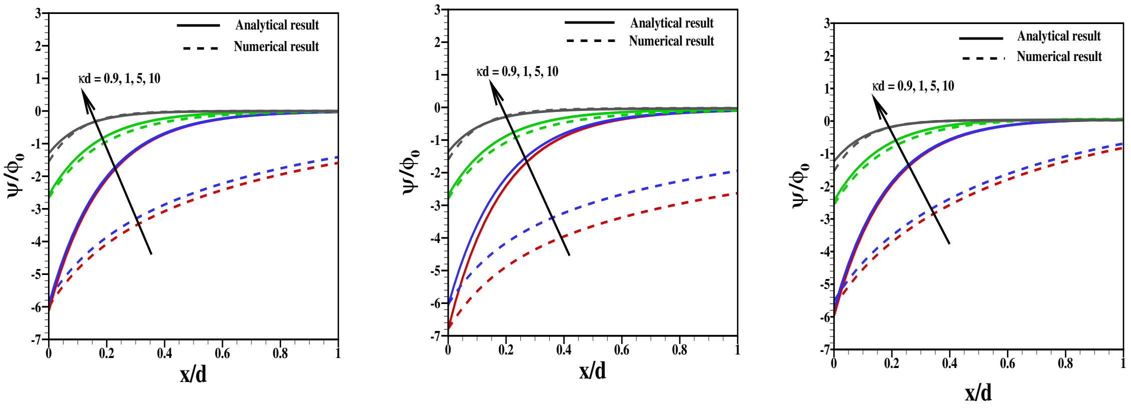

(a) (b) (c)

Figure S3: The analytical (presented by solid lines) and numerical results (represented by dashed lines) for scaled potential (ψ/ϕ_0), scaled by $\phi_0 = k_B T/ze$ is shown as a function of scaled spatial coordinate x/d for various values of κd (= 0.9, 1, 5, 10). We have shown the results when (a) $N = 0$, (b) $Z = -1$, $N = 10$ mM, (c) $Z = 1$, $N = 10$ mM. The results are shown for the fixed values of $\sigma = -25$ mC/m^2 and $d = 10$ nm.

In addition to the electrostatic potential, we have further shown a comparison of numerical results for scaled electrophoretic mobility with the deduced analytical results given in expression (34) appearing in main MS. The results in Fig S4 are presented for scaled electrophoretic mobility as a function of κd for three different cases, e.g., uncharged PEL, negatively charged PEL as well as positively charged PEL. The results are presented for fixed values of other model parameters. As expected we observed a closed agreement in numerical results with the approximate results for electrophoretic mobility for smaller values of κd.

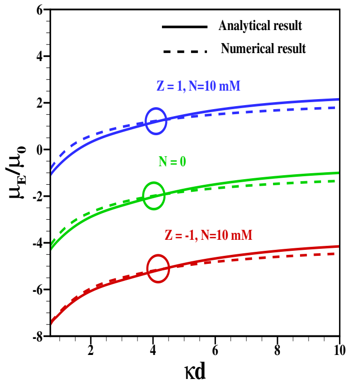

Figure S4: Scaled electrophoretic mobility μ_E/μ_0 is shown as a function of κd considering three different cases for PEL charge (e.g., uncharged PEL with $N = 0$, negatively charged PEL with $Z = -1$, $N = 10$ mM, and positively charged PEL with $Z = 1$, $N = 10$ mM). The results are shown for the fixed values of $r = 6$ Å, $\sigma = -25$ mC/m^2, $\beta = 1$ nm and $d = 10$ nm. Solid lines refers the deduced analytical results and dashed lines refers the numerical results for scaled electrophoretic mobility.

.
